# The genome of the versatile nitrogen fixer *Azorhizobium caulinodans *ORS571

**DOI:** 10.1186/1471-2164-9-271

**Published:** 2008-06-04

**Authors:** Kyung-Bum Lee, Philippe De Backer, Toshihiro Aono, Chi-Te Liu, Shino Suzuki, Tadahiro Suzuki, Takakazu Kaneko, Manabu Yamada, Satoshi Tabata, Doris M Kupfer, Fares Z Najar, Graham B Wiley, Bruce Roe, Tim T Binnewies, David W Ussery, Wim D'Haeze, Jeroen Den Herder, Dirk Gevers, Danny Vereecke, Marcelle Holsters, Hiroshi Oyaizu

**Affiliations:** 1 Laboratory of Plant Biotechnology, Biotechnology Research Center, University of Tokyo, Tokyo 113-8657, Japan; 2 Center for Information Biology and DNA Data Bank of Japan, National Institute of Genetics, Mishima 411-8540, Japan; 3 Department of Plant Systems Biology, Flanders Institute for Biotechnology (VIB), 9052 Gent, Belgium; 4 Department of Molecular Genetics, Ghent University, 9052 Gent, Belgium; 5 Kazusa DNA Research Institute, Chiba 292-0818, Japan; 6 Department of Chemistry and Biochemistry, University of Oklahoma, Norman, OK 73019-3051, USA; 7 Center for Biological Sequence Analysis, Technical University of Denmark, 2800 Lyngby, Denmark; 8 Department of Civil and Environmental Engineering, Massachusetts Institute of Technology, Cambridge, MA 02139-4307, USA

## Abstract

**Background:**

Biological nitrogen fixation is a prokaryotic process that plays an essential role in the global nitrogen cycle. *Azorhizobium caulinodans *ORS571 has the dual capacity to fix nitrogen both as free-living organism and in a symbiotic interaction with *Sesbania rostrata*. The host is a fast-growing, submergence-tolerant tropical legume on which *A. caulinodans *can efficiently induce nodule formation on the root system and on adventitious rootlets located on the stem.

**Results:**

The 5.37-Mb genome consists of a single circular chromosome with an overall average GC of 67% and numerous islands with varying GC contents. Most nodulation functions as well as a putative type-IV secretion system are found in a distinct symbiosis region. The genome contains a plethora of regulatory and transporter genes and many functions possibly involved in contacting a host. It potentially encodes 4717 proteins of which 96.3% have homologs and 3.7% are unique for *A. caulinodans*. Phylogenetic analyses show that the diazotroph *Xanthobacter autotrophicus *is the closest relative among the sequenced genomes, but the synteny between both genomes is very poor.

**Conclusion:**

The genome analysis reveals that *A. caulinodans *is a diazotroph that acquired the capacity to nodulate most probably through horizontal gene transfer of a complex symbiosis island. The genome contains numerous genes that reflect a strong adaptive and metabolic potential. These combined features and the availability of the annotated genome make *A. caulinodans *an attractive organism to explore symbiotic biological nitrogen fixation beyond leguminous plants.

## Background

Biological nitrogen fixation is carried out by a limited number of prokaryotes that all possess a nitrogenase enzyme complex that reduces molecular dinitrogen to ammonia. Nitrogen-fixing bacteria can be divided in two major groups: free-living nitrogen fixers or diazotrophs that directly assimilate ammonia for growth and symbiotic nitrogen fixers that pass ammonia to a eukaryotic host and indirectly profit from nitrogen fixation by occupying a particular ecological niche or by supporting the population through better feeding. In the latter group, the symbiosis between leguminous crop plants and rhizobia is of great importance for agriculture. The term "rhizobia" is used for bacteria that induce the formation of new organs, nodules, on the roots of a specific legume host. Inside the nodule, rhizobia are internalized in plant cells where they differentiate into nitrogen-fixing bacteroids [for a recent review on legume nodulation, see [1]].

Nitrogen-fixing nodules typically occur on roots; however, some members of the subfamilies Papilionoideae (*Aeschenomyne *sp., *Sesbania *sp., and *Discolobium pulchellum*) and Mimosoideae (*Neptunia oleracea*) form stem-located, aerial nodules [2]. These legumes grow in waterlogged soils of tropical regions and are characterized by dormant, stem-located adventitious root primordia that can develop into stem nodules upon inoculation with an appropriate microbial partner. Although stem and root nodulation are similar, in the latter the nodular vascular system is connected to that of the stem via the vascular bundles of the adventitious root primordium [3].

A particularly well-studied case of stem nodulation occurs in *Sesbania rostrata *Brem. upon inoculation with the microsymbiont *Azorhizobium caulinodans *[4]. *S. rostrata*, a fast-growing annual shrub from the Sahel region of West-Africa, carries numerous adventitious root primordia that protrude through the stem cortex and epidermis, creating a circular fissure, where bacteria can invade and proliferate [5]. The growth properties and the high rate of nitrogen fixation of stem-nodulated plants make *S. rostrata *well fit as green manure in rice cultivation and, possibly, as a pioneer plant for wetland improvement [6].

The bacterium, isolated from stem nodules [4] and originally designated *Rhizobium *sp. strain ORS571, was renamed *Azorhizobium caulinodans *inspired by the stem (cauli-)nodulating capacity and by the diazotrophic properties of the strain (azo-rhizobium). Its host range for effective nodulation is very narrow: although nodulation of several *Sesbania *sp. has been reported, nitrogen-fixing nodules are formed only on *S. rostrata *and *S. punctata *[7]. *A. caulinodans *also induces Fix nodules on *Phaseolus vulgaris *and *Leucaena leucocephala *[8]. Two features distinguish *A. caulinodans *from other rhizobia: its taxonomic position and its dual capacity for free-living and symbiotic nitrogen fixation. The latter is exceptional [9] and implies a regulatory mechanism to either assimilate the ammonia or donate it to the plant in the symbiotic interaction. The first taxonomic study of *A. caulinodans *strain ORS571 [10] showed that it belongs to the *Rhodopseudomonas palustris *rRNA branch of purple bacteria, but that it is quite distinct from both *Rhodopseudomonas *and *Bradyrhizobium *spp. Based on numerical analysis of phenotypes, protein patterns, and DNA-DNA and DNA-rRNA hybridizations, *A. caulinodans *was considered as a separate genus with *Xanthobacter *as closest relative [11]. *Xanthobacter *sp. are diazotrophic bacteria found in diverse soil habitats and in association with rice (*Oryza sativa*) roots [12,13]. Comparison of 16S rRNA sequences indicated that *X. flavus *and *A. caulinodans *are strongly related [14].

Here, we present the genome sequence of the *A. caulinodans *strain ORS571 and discuss the annotation in function of the organism's biology with reference to comparative genomics. This information will stimulate the research on an organism that has real potential for novel applications in agriculture.

## Results

### Genome organization

**Figure 1:**
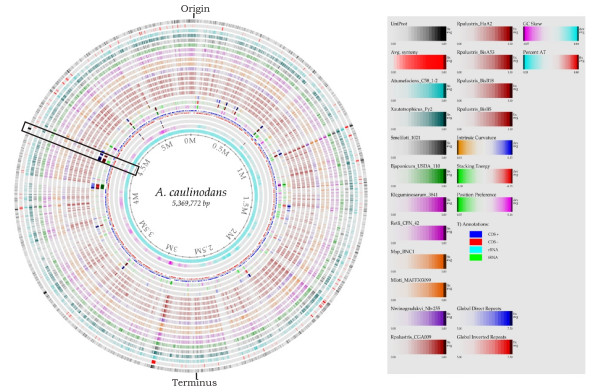
**Snapshot of the output generated after analysis of the *A. caulinodans *genome with the Genome Atlas tool**. The output is accessible as a web-based resource [17] that can can be used as a tool to zoom in on specific regions of interest. Hits within the UNIPROT database, a comparison at the protein level between 14 α-proteobacteria and *A. caulinodans*, and the synteny between these genomes, the genome annotation, and structural features are represented. The origin and terminus of replication are indicated and the symbiotic region is boxed. From the outer to the inner circle: circle 1, protein hits in the UNIPROT database; circle 2, synteny between 15 α-proteobacterial genomes; circle 3, *Agrobacterium tumefaciens *C58; circle 4, *Xanthobacter autotrophicus *Py2; circle 5, *Sinorhizobium meliloti *1021; circle 6, *Bradyrhizobium japonicum *USDA 110; circle 7, *Rhizobium leguminosarum *3841; circle 8, *Rhizobium etli *CFN42; circles 9 and 10, *Mesorhizobium loti *strains BNC1 and MAFF303099, respectively; circle 11, *Nitrobacter winogradskyi *Nb225; circles 12, 13, 14, 15, and 16, *Rhodopseudomonas palustris *strains CGA009, HaA2, BisA53, BisB18, and BisB5, respectively; circle 17, intrinsic curvature; circle 18, stacking energy; circle 19, position preference; circle 20, genome annotation; circle 21, global repeats; circle 22, inverted repeats; circle 23, GC skew; circle 24, percent AT. The structural DNA parameters in circles 21 and 22 relate to the occurrence of repeats that might indicate inserted sequences, and circles 18 and 19 designate the accessibility and flexibility of the DNA as a measure for the capacity to interact with proteins.

**Figure 2:**
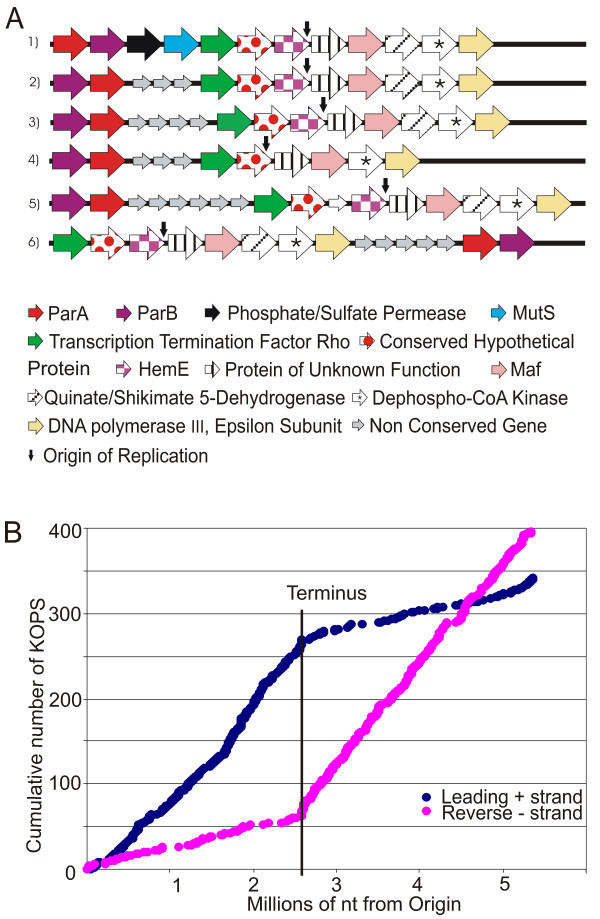
**Predicted position of the origin of replication**. A. Conservation of a cluster of 12 genes located around the origin of replication in several α-proteobacteria: (1) *Azorhizobium caulinodans*, (2) *Agrobacterium tumefaciens*, *Sinorhizobium meliloti*, *Rhizobium leguminosarum*, and *Rhizobium etli*, (3) *Mesorhizobium loti*, *Brucella abortus*, and *Brucella suis*, (4) *Bradyrhizobium japonicum *and *Rhodopseudomonas palustris*, (5) *Caulobacter crescentus*, and (6) *Xanthobacter autotrophicus*. The putative proteins and the origin of replication are indicated. B. Cumulative distribution in forward and reverse orientation of the 8-base KOPS motif 5'-GGGNAGGG-3' in the genome of *A. caulinodans*. The orientation of this motif is strongly biased toward *dif *sites at the terminus of replication (Terminus).

Sequencing of the genome of *A. caulinodans *strain ORS571 (hereafter designated *A. caulinodans*) revealed a single circular chromosome of 5,369,772 base pairs [15]. Relevant genome features generated with the BLASTatlas tool [16] are presented in Figure 1 and can be viewed in detail as a web-based resource [17]. The putative origin of replication was predicted based on the position of a GC skew shift (Figure 1) [18] and coincided with the occurrence of a gene cluster typically associated with origins of replication in circular chromosomes of α-proteobacteria (Figure 2A) [19]. The specific distribution and orientation of the FtsK Orienting Polar Sequences (KOPS) motif 5'-GGGNAGGG-3', which is involved in loading the FtsK DNA translocase and directing it to the replication terminus in α-proteobacteria [20], confirmed the predicted location of the origin between AZC_4717 and AZC_0001 (Figure 2B).

**Figure 3:**
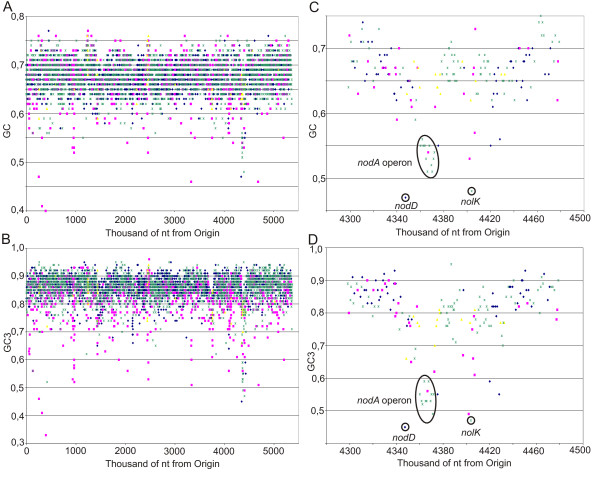
**Nucleotide composition of the *A. caulinodans *genome**. The GC and GC3 contents for each open reading frame were calculated and positioned on the genome. Every gene was classified in one of four classes: orphans, genes of the *A. caulinodans *genome without homolog in other bacteria of the data set (44 genomes) (red squares); singletons, genes with one representative in *A. caulinodans *and homologs in the data set (green stars); phage- or integrase-related genes (yellow triangles); duplicated genes with more than one paralog in the *A. caulinodans *genome (blue diamonds). GC (A) and GC3 (B) distribution across the genome; GC (C) and GC3 (D) distribution across the symbiotic region. Circles (C and D) indicate the location of the three *nod *loci.

**Figure 4:**
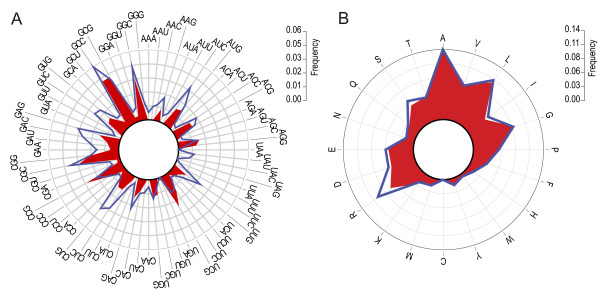
Codon (A) and amino acid (B) usage in the *A. caulinodans *genome (red) and the symbiotic region (blue).

Although the overall GC content of the *A. caulinodans *genome is 67% and the average GC incidence at the third position of the codon (GC3) is 85%, the chromosome has many islands of varying size with different GC (Figure 3A) and GC3 contents (Figure 3B). In accordance with the overall high GC content, the codon usage is shifted toward GC-rich codons (Figure 4A) and, consequently, GC-coded amino acids are overrepresented (Figure 4B).

Combined computer prediction and similarity searches (Methods) revealed 4717 protein-encoding genes with an average coding density of one gene in every 1123 bp (89%). With the BLASTP program (Methods), the amino acid sequences were compared with the sequences in the nonredundant protein database at NCBI. A putative function could be assigned to 3588 genes (76.1%), 954 genes (20.2%) were similar to hypothetical genes, and the remaining 175 (3.7%) had no significant similarity to any registered gene (Figure 1; Table 1; Additional file 1).

**Table 1:** Overview of the functional categories of proteins present in the *A. caulinodans *genome according to the classification of Riley [26].

Functional classification	No.^a^	%^b^
Amino acid biosynthesis	132	2.8
Biosynthesis of cofactors, prosthetic groups and carriers	159	3.4
Cell envelope	174	3.7
Cellular processes	200	4.3
Central intermediary metabolism	161	3.4
Energy metabolism	303	6.4
Fatty acid, phopholipid, and sterol metabolism	136	2.9
Purines, pyrimidines, nucleosides, and nucleotides	66	1.4
Regulatory function	384	8.1
DNA replication, recombination and repair	79	1.7
Transcription	49	1.0
Signal transduction	39	0.8
Translation	227	4.8
Transport and binding proteins	714	15.2
Other categories	765	16.2
Hypothetical protein	954	20.2
Unknown protein	175	3.7

Total	4717	100.0

^a ^Number of proteins that belong to a specific class.

^b ^Percentage of total proteins that belong to a specific class.

Three rRNA clusters are ordered as 5S-23S-16S (located between the protein-coding genes AZC_0613-AZC_0614, AZC_4195-AZC_4196, and AZC_4435-AZC_4436) and all have an insertion of a tRNA-Ile and a tRNA-Ala between the 16S and 23S genes. A total of 53 tRNA genes representing 44 tRNA species for all 20 amino acids were assigned by sequence similarity and computer prediction with the tRNAscan-SE program [21]. Most of the tRNA genes are dispersed on the genome and are probably transcribed as single units. Thirty out of 57 ribosomal protein genes occur in a cluster (AZC_2529-AZC_2559), whereas the others are scattered over the genome (Additional file 1).

### Phylogeny and comparative genomics

**Figure 5:**
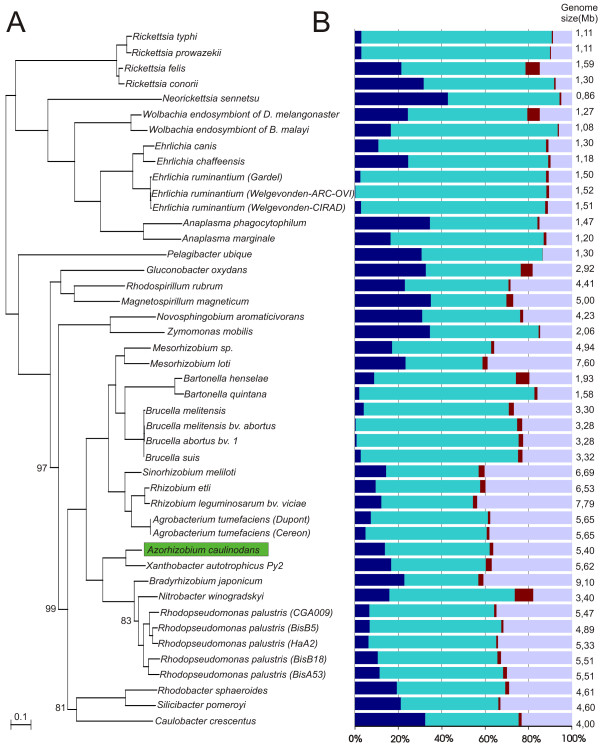
**Maximum-likelihood tree and prevalence of duplicated, singleton, and orphan genes within 45 complete α-proteobacterial genome sequences**. A. Unrooted maximum-likelihood tree based on 108 conserved protein sequences (for construction of the tree and references to the genomes, see Methods and Additional file 2, respectively). Bootstraps are 100, unless indicated otherwise. B. Percentual presence of the four gene categories: orphans (dark blue); singletons (cyan); phage- or integrase-related genes (red); and duplicated genes or paralogs (light blue). Genome sizes are indicated on the right.

For phylogenetic analysis (Methods), the genomes of *A. caulinodans *and of 44 α-proteobacteria were compared (Additional file 2). The data set was assembled based on the available complete genome sequences (closure date August 15, 2007) and ecological or phylogenetic relatedness. The resulting maximum-likelihood tree (Figure 5A) showed a great concordance with α-proteobacterial trees based on complete 16S rRNA genes [22] or sets of protein families [23]. Our analysis placed *A. caulinodans *closest to *X. autotrophicus*, *Nitrobacter winogradskyi*, *Rhodopseudomonas palustris*, and *Bradyrhizobium japonicum*, consistent with previous taxonomic studies [9,10,13]. With *A. caulinodans *as a reference genome, a graphical representation of the BLAST hits of the proteins encoded by the genomes of the 13 closest relatives was generated with the BLASTatlas tool (Figure 1) [16,17].

For a broader view of the gene relationships, the occurrence and organization of the proteins encoded by these 45 genomes were evaluated (Methods). Each gene of a total data set of 146,315 was classified in one of four groups: orphans, genes without homologs in other bacteria of the data set; singletons, genes with one representative in the genome and homologs in other genomes; phage or integrase-related genes; and duplicated genes or paralogs with more than one paralog in the genome. The distribution of each of these categories differed in the surveyed genomes (Figure 5B). Paralog representation ranged from 5% for the *Neorickettsia sennetsu *strain *Miyayama *(genome size 0.86 Mb) to 44% for *Rhizobium leguminosarum *bv. *vicae *(strain 3841) (genome size 7.79 Mb), whereas *A. caulinodans *had 36% paralogs (genome size 5.37 Mb). The data confirmed the observation that the number of paralogs strongly correlates with the genome size in a linear regression [24].

Altogether, these analyses demonstrate that currently *X. autotrophicus *is the closest sequenced relative of *A. caulinodans*. However, a comparison of the genomes with the ARTEMIS comparison tool [25] revealed a very low degree of synteny (Additional file 3). Although short sequence stretches are conserved, extensive rearrangements have taken place. The occurrence of four prophages and numerous transposases in the *A. caulinodans *genome suggests a high genome plasticity. In *A. caulinodans*, 1412 proteins have no counterpart in *X. autotrophicus *of which 544 (38%) are catalogued as unknown or hypothetical (Additional file 4). In the remaining group of functionally classified proteins, 46% have GC and GC3 contents different from the genome averages, suggesting recent acquisition.

### Functional protein classes and metabolic pathways

The putative protein-encoding genes were ordered into 17 classes [26] (Table 1) and the metabolic potential of *A. caulinodans *was analyzed with the PathoLogic tool of the BioCyC/MetaCYC suite [27].

These analyses revealed the presence of many regulatory genes (8%) and several RNA polymerase σ factors, among which two household σ^70 ^factors (AZC_3643 and AZC_4253), two σ^54 ^factors (AZC_2924 and AZC_3925; see below), and five σ factors of the extracytoplasmic subclass (AZC_0389, AZC_1202, AZC_2427, AZC_2453, and AZC_3238), implying responsiveness to many environmental triggers. As *A. caulinodans *is a motile bacterium, a large gene cluster is present (AZC_0615-AZC_0666) for the formation of a type-III flagellum. A significant number of chemotaxis genes predicts the capacity to respond to a wide array of molecules (Additional file 5). While no complete quorum sensing system could be detected, the presence of no less than five LuxR-type response regulators suggests that *A. caulinodans *has the potential to listen in on acyl-homoserine lactone-mediated communication in its surroundings.

A variety of encoded proteins might offer protection against toxic compounds in the environment (Additional file 6). Examples are two cytochrome P450 monooxygenases and pathways to degrade or modify plant-derived molecules, such as protocatechuate, and xenobiotics, such as cyanate, 1,4-dichlorobenzene, octane, and gallate. Several multidrug efflux pumps, antibiotic-modifying enzymes, and heavy metal translocation systems probably confer resistance to deleterious compounds. The production of the siderophores enterobactin and aerobactin might guarantee iron acquisition from the surroundings.

The surface of bacteria is important for recognition, attachment, and colonization during the interaction with a host. Exopolysaccharides and lipopolysaccharides are involved in nodulation as protective compounds against defense molecules generated by the plant and as communication signals [28-30]. Other functions could relate to surface structures, important for interaction with the host (Additional file 7), e.g. putative adhesion proteins, antigens, and 29 genes that code for proteins with GGDEF/EAL domains. The latter typically play a role in the transition from a motile planktonic form to a sessile biofilm by controlling the formation and degradation of the secondary messenger cyclic di-GMP [31]. Hormones also play an important role in plant-microbe interactions. Both a structural (AZC_0267) and a regulatory gene (AZC_0266) mediating degradation of the ethylene precursor 1-aminocyclopropane-1-carboxylate, are present in the genome.

Over 15% of the genes are dedicated to "transport and binding", of which more than 50% belong to the ATP-binding-cassette (ABC) transporter class. With 118 complete systems (consisting of a solute-binding protein, a permease, and an ABC component for the uptake systems, or an ABC component and a permease for the export systems), and numerous orphan subunits scattered over the genome, the transporter complement of *A. caulinodans *equals that of many other soil bacteria. These high-affinity transport systems are dedicated to the uptake of peptides, amino acids, sugars, polyamines, siderophores, nitrate/sulfonate/bicarbonate, or C4-dicarboxylate and many unknown substrates (Additional file 8). Accordingly, catabolic pathways are predicted for compounds, such as amino acids (including citrulline and ornithine), glucuronate, galactonate, galactarate, gluconate, quinate, L-idonate, creatinine, and 4-hydroxymandelate. Sugars, such as glucose, fructose, sucrose, ribose, xylose, xylulose, and lactose are not metabolized by *A. caulinodans*; instead, dicarboxylic acids are used as primary carbon source [10], as reflected by the presence of multiple C4-dicarboxylic acid transport systems. The occurrence of 16 putative alcohol dehydrogenase genes suggests that ethanol could be a major carbon source under flooded conditions. *A. caulinodans *is also capable of oxidizing hydrogen, an obligatory by-product of the nitrogenase, and the required *hup, hyp*, and *hox*A genes are located in a large gene cluster (AZC_0594-AZC_0613) [32]. Encoded energy metabolism pathways include glycolysis, Entner-Doudoroff, and TCA cycle. The absence of a gene encoding phosphofructokinase indicates the lack of a functional Emden-Meyerhof pathway.

### Nitrogen fixation and related functions

Table 2 lists the genomic position of *A. caulinodans *genes related to free-living and symbiotic nitrogen fixation. These genes code for known functions, such as formation of the nitrogenase, assembly and stabilization of the complex, synthesis of the MoFe cofactor and the FeS clusters, electron transport, ammonium assimilation, and regulation of gene expression by nitrogen and oxygen, but also for proteins whose exact role await experimental confirmation. Several *nif *genes occur in more than one copy and are scattered over the genome as solitary loci or clusters of varying size with GC and GC3 contents matching the averages of the genome (Additional file 1). The NifH phylogeny was congruent with the phylogenetic relationships based on 16S rRNA [33] or on core protein families [Figure 5A]. The same holds true for the other genes listed in Table 2 (data not shown).

**Table 2:** Nitrogen fixation-related genes

Identifier	Gene	Description
AZC_0344	*pts*N	PTS IIA-like nitrogen-regulatory protein
AZC_1036	*nif*X	Nitrogenase MoFe cofactor biosynthesis
AZC_1037	*nif*N	Nitrogenase MoFe cofactor biosynthesis
AZC_1038	*nif*E	Nitrogenase MoFe cofactor biosynthesis
AZC_1039	*nif*K	Nitrogenase MoFe protein β-chain
AZC_1040	*nif*D	Nitrogenase MoFe protein α-chain
AZC_1041	*nif*H	Dinitrogenase reductase
AZC_1049	*nif*A	Transcriptional activator
AZC_1601	*gln*A	Glutamine synthetase
AZC_1602	*gln*B	Nitrogen regulatory protein
AZC_2280	*ntr*B*/ntr*Y	Signal transduction histidine kinase
AZC_2924	*rpo*F	RNA polymerase σ^54 ^factor
AZC_3080	*nfr*A	Translation regulator of *nif*A
AZC_3083	*ntr*X	Transcriptional regulator
AZC_3084	*ntr*Y	Signal transduction histidine kinase
AZC_3086	*ntr*C	Transcriptional regulator
AZC_3087	*ntr*B	Signal transduction histidine kinase
AZC_3088	*nif*R3	Nitrogen assimilation-regulatory protein
AZC_3410	*nif*U	Mobilization of Fe for Fe-S cluster synthesis and repair
AZC_3411	*nif*S	Nitrogenase cofactor synthesis protein
AZC_3412	*fix*U	Unknown function
AZC_3414	*nif*B	Fe and S donor for MoFe cofactor biosynthesis
AZC_3420	*nif*Z	Unknown function
AZC_3443	*nif*H	Dinitrogenase reductase
AZC_3444	*nif*Q	Nitrogenase MoFe cofactor biosynthesis
AZC_3446	*nif*W	Nitrogenase-stabilizing/protective protein
AZC_3447	*fix*A	Electron-transferring flavoprotein oxidoreductase
AZC_3448	*fix*B	Electron-transferring flavoprotein
AZC_3449	*fix*C	Electron-transferring flavoprotein oxidoreductase
AZC_3450	*fix*X	Ferredoxin protein
AZC_3925	*rpo*N	RNA polymerase σ^54 ^factor
AZC_4523	*cyt*N*/fix*N	Cytochrome c oxidase subunit 1
AZC_4524	*cyt*O*/fix*O	Cytochrome c oxidase subunit 2
AZC_4525	*cyt*Q*/fix*Q	Cytochrome c oxidase subunit 3
AZC_4526	*cyt*P*/fix*P	Cytochrome c oxidase subunit 4
AZC_4527	*fix*G	Assembly and stability of the FixNOQP complex
AZC_4528	*fix*H	Assembly and stability of the FixNOQP complex
AZC_4653	*fix*K	Transcriptional activator
AZC_4654	*fix*L	Sensor protein
AZC_4655	*fix*J	Transcriptional regulatory protein

The transcriptional activator NifA (AZC_1049) acts together with a σ^54 ^factor RpoN (AZC_3925) to control the *nif/fix *gene expression [34]. Nitrogen regulation of *nifA *expression is under control of the NtrBC (AZC_3086-AZC_3087) and NtrYX (AZC_3083-AZC_3084) two-component systems [35,36] that respond to the intracellular and extracellular nitrogen status, respectively. The expression of these two loci depends on a hypothetical σ^54 ^factor RpoF [34], which presumably corresponds to AZC_2924. Oxygen control of *nif*A expression is mediated by FixLJ (AZC_4654 and AZC_4655) [37], and the transcription factor FixK (AZC_4653) [38]. The *nif*A gene is further controlled at the transcriptional level by a LysR-type regulator [39] and at the translational level by the *nrf*A gene product (AZC_3080) [40]. FixK also activates transcription of the *cyt*NOQP operon (AZC_4523-AZC_4526), encoding the high-affinity terminal oxidase cytochrome *cbb3 *that is induced under microaerobiosis [41,42]. Mutants in *cyt*NOQP still fix nitrogen under free-living conditions, suggesting the occurrence of another terminal oxidase [41,43]. The survey of the genome excluded the presence of a second cytochrome *cbb3 *complex, but revealed two cytochrome *bd *complexes (AZC_1353-AZC_1354 and AZC_3759-AZC_3760).

### A symbiosis region

**Figure 6:**
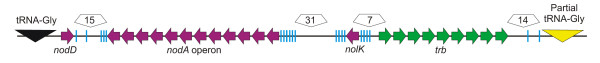
**Schematic representation of the symbiosis region**. Genes described in the text are indicated by arrows; the others are not individually represented, but their number is specified in the pentagons. The symbiotic region is flanked by tRNA-Gly (triangles) and interspersed by multiple transposases and integrases (blue lines). Genes in the *nod*A operon are *nod*ABCSUIJZ*noe*CHOP and genes in the *trb *operon are *trb*BCDEJLFGI.

A region of 87.6 kb, delimited by a Gly-tRNA (position 4346061) and an integrase (AZC_3882) and flanked by direct repeats (Figure 6), is characterized by an overall lower GC (Figure 3C) and GC3 contents (Figure 3D) than the genome averages, and a different preferential codon usage (Figure 4A). No less than 18 putative transposases and three integrases are present, suggesting a complex history of horizontal gene transfer events. The region contains the three *nod *loci that are involved in the synthesis and secretion of the lipochitooligosaccharide Nod factors (NFs) [44], but also genes related to chemotaxis, amino acid uptake, and a putative type-IV secretion system (Additional file 1).

The three *nod *loci are not adjacent and have a GC content lower than that of the surrounding sequences (Figure 3C). The shifts in GC content correspond to the location of repeated elements that are flanked by insertion sequences or tRNAs (Figures 1 and 6). The constitutively expressed *nod*D gene (AZC_3792) [45,46] codes for a LysR-type regulator that activates transcription of the two other flavonoid-inducible *nod *loci. The inducible operon *nod*ABCSUIJZ*noe*CHOP (AZC_3818-AZC_3807) [47-49] encodes most of the enzymatic machinery for NF backbone synthesis, decoration, and secretion. The biochemical role of these proteins has been extensively described, except for the last four open reading frames *noe*CHOP that are involved in NF arabinosylation and are still under study. Based on similarity with proteins involved in arabinosylation of the cell wall in *Mycobacterium tuberculosis*, *noe*C (AZC_3810), *noe*H (AZC_3809), and *noe*O (AZC_3808) might encode the synthesis of a D-arabinose precursor [50-52]. The third locus encodes the inducible *nol*K gene responsible for GDP-fucose synthesis for NF decoration (AZC_3850) [53,54].

The symbiosis region also contains two conjugation-related gene clusters with GC and GC3 contents comparable to the genome averages. The cluster AZC_3844-AZC_3826 – flanked by two transposases – consists of *repA *and genes encoding conjugal transfer, partition, and plasmid stabilization proteins (Additional file 1). In the cluster AZC_3856–3877, flanked by a transposase and an integrase, genes are found that are homologous to the *trb*BCDEJLFGI genes, a type-IV secretion system involved in conjugative transfer of the tumor-inducing plasmid in *Agrobacterium tumefaciens *[55] (Figure 6).

The genome annotation indicates the presence of a few additional nodulation-related genes outside of the symbiosis region (Additional file 1). Two response regulators (AZC_1361 and AZC_2281) homologous to *nod*W genes of *Bradyrhizobium japonicum *and part of a two-component signal transduction system might be involved in the response to host-exuded flavonoids [56]. A *nod*T-related gene (AZC_3288) [57] might act as the outer-membrane component in NF secretion together with the inner-membrane NodIJ proteins. None of these four potential nodulation genes has a different GC or GC3 content, in contrast to the *nod *genes of the symbiosis region.

## Discussion

*Azorhizobium caulinodans *is a member of the α-proteobacteria, a group with diverse genome architectures. Several plant-associated representatives, such as *Agrobacterium *and *Sinorhizobium*, have quite considerable genomes and large circular or linear plasmids. In contrast, *A. caulinodans *has a single circular chromosome of 5.37 Mb and no auxiliary replicons. The GC content and the coding density are in range with other rhizobia and soil bacteria. *A. caulinodans *is a motile, nitrogen-fixing, hydrogen-oxidizing, aerobic bacterium with a preference for organic acids as carbon source. This lifestyle is reflected in the metabolic pathways and in clusters for flagellum synthesis, motility, and chemotaxis. A high number of genes are dedicated to transport and regulation, indicating that a wide range of substrates can be taken up, but that the pathways are tightly regulated to limit the metabolic burden. Besides the well-described role of surface polysaccharides during plant-microbe interactions, the genome of *A. caulinodans *encodes functions that might be involved in biofilm formation, possibly facilitating the interaction with a host. Ongoing functional analysis will undoubtedly reveal new players in the ecology of the dual lifestyle of *A. caulinodans *[58,59].

Genome analysis combined with phylogenetic studies has shed new light on bacterial evolution and taxonomy. Core functions can be identified that are highly conserved between related groups, but that may acquire individual characteristics through accessory genes [60]. Analysis of a family of core proteins and 16S rDNA sequence comparison revealed that the closest relative of *A. caulinodans *is *Xanthobacter autotrophicus*. *Xanthobacter *sp. are free-living nitrogen fixers and the *nif *and *fix *genes can thus be considered part of the core functions of the *Azorhizobium-Xanthobacter *group. The major difference in the lifestyle of both organisms is that *A. caulinodans *has acquired the ability to establish a symbiosis with *S. rostrata*.

The nodulation capacities are related to the presence of a symbiosis region with distinct GC and GC3 contents and codon usage. The association with tRNA loci, which presumably act as targets for the integration of foreign DNA, and multiple transposons suggest a high plasticity of this region, as reflected in its composition. The symbiosis region contains three subclusters related to nodulation, *nod*ABCSUIJZ*noe*CHOP, *nod*D, and *nol*K that are flanked by sequences suggestive of independent horizontal acquisition. The repeated elements could be the relics of insertion elements that once played a role in the evolution of the *A. caulinodans *nodulation genes that have all the characteristics of archetypal accessory genes.

To study the evolution of *nod *genes, *A. caulinodans *forms an interesting case. The azorhizobial *nod *genes are only distantly related to their counterparts in other rhizobia. Phylogenetic comparisons demonstrated that the *nod*A and *nod*C genes from rhizobia that nodulate temperate legumes (e.g. *S. meliloti*, *R. leguminosarum *bv. *viciae *and bv. *trifolii*, and *R. galegae*) are grouped together and the genes from rhizobia that nodulate tropical legumes (e.g. *B. japonicum*, *B. elkanii*, *R. loti*, *R. tropici*, and *R. etli*) form a second cluster [61,62]. However, the *nod*A, *nod*B, and *nod*C genes of *A. caulinodans *belong neither to the tropical nor the temperate clusters [62,63]. Also, the genetic distance between the *nod*SU genes of *A. caulinodans *and their counterparts in other rhizobia is much greater than the mutual genetic distance between the *nod*SU genes of these rhizobia [8]. The organization of the *nod*ABCSUIJ genes in *A. caulinodans *resembles the situation in *B. japonicum*, but the upstream and downstream regions are different [64,65].

At present we do not know the origin of the symbiotic genes of *A. caulinodans*. The Rhizobiaceae, which have been historically considered a true family in phylogenetic terms, now seem a rather diverse group of bacteria, including *Methylobacterium*, *Ralstonia*, and *Burkholderia *that share variant, relatively recently acquired, symbiotic gene clusters. Possibly, the *A. caulinodans nod *genes are derived from unexplored rhizobia or even from obligate endophytes. Undoubtedly, the recent and ongoing explosion in meta-genomic projects will provide more insight into the origin of the nodulation functions.

## Conclusion

Extension of symbiotic nitrogen fixation to non-legume cereal plants is a challenging long-standing goal. Especially, there is a growing interest in nitrogen-fixing organisms that could establish an endophytic, beneficial relation with important crops, such as rice and wheat (*Triticum aestivum*). Interestingly in this context, the occurrence of *A. caulinodans *has been reported in intercellular infection pockets located in the cortex of roots of *Arabidopsis thaliana *and wheat [66]. In fields where *S. rostrata *and rice are grown as rotation crops, *A. caulinodans *seems to survive very well in the rhizosphere of the rice plants and in the soil [67]. Moreover, the bacterium invades emerging lateral roots of rice, and rice seedlings inoculated with *A. caulinodans *have a high nitrogenase activity [68]. Finally, *A. caulinodans *fixes nitrogen under relatively high oxygen tension as a free-living organism, invades the host via cracks, and establishes intercellular colonies. Altogether, these features might be advantageous for primary infection of nitrogen-starved root systems and highlight the potential of *A. caulinodans *as a candidate model organism. The genome sequence data provide new opportunities for exploring the regulatory aspects of *Azorhizobium *nitrogen fixation and the essential features that implement the ability for endosymbiosis.

## Methods

### DNA sequencing

The nucleotide sequence of the entire genome of *A. caulinodans *ORS571 was determined by the whole-genome shotgun strategy method. For shotgun cloning, DNA fragments of 2 to 3 kb were cloned into the *Hin*cII site of pUC118. For gap closing, the pCC1Fos vector (Epicentre, Madison, WI, USA) was used, and approximately 35-kb clones were prepared. The accumulated sequence files were assembled with the Phrap program [69]. A total of 71,424 random sequence files corresponding to approximately 7.7 genome equivalents were assembled to generate draft sequences. Finishing was carried out by visual editing of the sequences, followed by gap closing, and additional sequencing to obtain sequence data with a Phred score of 20 or higher [70,71]. The integrity of the final genome sequence was assessed by comparing the insert length of each fosmid clone with the computed distance between the end sequences of the clones. The end sequence data facilitated gap closure as well as accurate reconstruction of the entire genome. The final gaps in the sequences were filled by the primer walking method. A lower threshold of acceptability for the generation of consensus sequences was set at a Phred score of 20 for each base. The nucleotide sequence is available in the DDBJ/EMBL/GenBank databases under the accession number AP009384.

### Structural and functional annotation

Coding regions were assigned by a combination of computer prediction and similarity search. Briefly, the protein-coding regions were predicted with the Glimmer 2.02 program [72] and all regions equal to or longer than 90 bp were translated into amino acid sequences that were subjected to similarity searches against the nonredundant protein database at NCBI with the BLASTP program [73]. In parallel, the entire genomic sequence was compared with those in the nonredundant protein database with the BLASTX program [73] to identify genes that had escaped prediction and/or were smaller than 90 bp, especially in the predicted intergenic regions. For predicted genes without sequence similarity to known genes, only those equal to or longer than 150 bp were considered as candidates. Functions were assigned to the predicted genes based on sequence similarity of their deduced products to that of genes of known function. For genes that encode proteins of 100 amino acid residues or more, an E-value of 10^-20 ^was considered significant, whereas a higher E-value was significant for genes encoding smaller proteins (E-value treshold of 10^-10^). Genes for structural RNAs were assigned by similarity search against the in-house structural RNA database that had been generated based on the GenBank data. tRNA-encoding regions were predicted by the tRNAscan-SE 1.21 program [21] in combination with the similarity search.

MetaCyc analysis [27,74] detected 229 metabolic pathways, containing 1037 reaction steps. To assess the presence or absence of a metabolic pathway and to decrease the likelihood of being misled by the many enzymes that are shared among multiple pathways, the analysis was emphasized on the presence of enzymes that are unique to a pathway.

### Construction of a phylogenetic tree

The Maximum-likelihood tree was based on 108 core proteins of 45 α-proteobacteria [23] whose sequence data and annotation files were available and downloaded from the NCBI Microbial Genome Resource database [75]. The set of core genes was determined by an all-against-all BLAST at protein level. Best reciprocal hits were selected, taking into account a cut-off value defined as 20% similarity and an overlap of at least 150 amino acids. Only proteins present in all 45 genomes as single copy were considered as "core proteins" and used to construct the phylogenetic tree. The total alignment contained 32,327 amino acids. The tree was constructed with the Phyml program [76] and a WAG substitution model [77] and 100 bootstrap replicates were run. Unless indicated otherwise, bootstraps are 100 (Figure 5A).

## Authors' contributions

K-BL contributed to the sequence determination and carried out the annotation of the genes. PDB analyzed the data generating the figures and the manuscript. TA, C-TL, SS, and TS contributed to the sequence determination. TK, MY, and ST annotated the gene sequences. DMK, FZN and GBW sequenced the DNA and assembled and interpreted the DNA sequence data. BR participated in the design and coordination of the study, in particular the original conception, the DNA sequencing, and subsequent DNA analysis. TTB and DWU constructed and analyzed the BLASTatlas of *Azorhizobium*. WD'H assisted in the preparation of the *Azorhizobium *genomic DNA and analysis of genetic data. JDH contributed to the MetaCyc analysis. DG carried out the evolutionary analysis of the genome. DV and MH interpreted the data and figures and wrote the article. HO contributed to sequence determination and gene annotation. All authors have read and approved the final version of the manuscript.

## Supplementary Material

Additional file 1 **Overview of the genes, their properties, and translation products present in the genome of *A. caulinodans***. From left to right: unique ID for each *A. caulinodans *gene; indication of the gene function based on homology; functional classification of genes according to Riley [26]; GC content at the third position of codons in percent; GC content of a gene in percent; start/stop, position of start codon or stop codon of a gene on the plus or minus strand, respectively; stop/start, position of stop codon or start codon of a gene on the plus or minus strand, respectively; strand, coding sequence on the plus or minus strand; protein sequence. Click here for file

Additional file 2 **Overview of the properties of the 45 α-proteobacterial genomes of the data set**. From left to right: bacterial strain; type of replicon; NCBI database identification code; size in Mbp; GC content of the replicon; number of proteins encoded by the replicon; number of structural RNA elements on the replicon. Click here for file

Additional file 3 **Whole genome comparison of *A. caulinodans *ORS571 and *Xanthobacter autotrophicus *Py2 using the ARTEMIS Comparison Tool **[25]. Red and blue lines connect similar sequences and similar sequences that are inverted between strains, respectively. Click here for file

Additional file 4 **Overview of genes present in the *A. caulinodans *ORS571 genome and absent in the *Xanthobacter autotrophicus *Py2 genome**. From left to right: unique ID for each *A. caulinodans *gene; indication of the gene function based on homology; GC content at the third position of codons in percent; GC content of a gene in percent. Click here for file

Additional file 5 **Chemotaxis and motility genes in the *A. caulinodans *genome**. From left to right: unique ID for each *A. caulinodans *gene; indication of the gene function based on homology; functional classification of genes according to Riley [26]; GC content at the silent third base of codons in percent; GC content of a gene in percent. Click here for file

Additional file 6 **Putative detoxification genes in *A. caulinodans***. From left to right: unique ID for each *A. caulinodans *gene; indication of the gene function based on homology; functional classification of genes according to Riley [26]; GC content at the third position of codons in percent; GC content of a gene in percent. Click here for file

Additional file 7 **Genes encoding surface-associated components in the *A. caulinodans *genome**. From left to right: unique ID for each *A. caulinodans *gene; indication of the gene function based on homology; GC content at the third position of codons in percent; GC content of a gene in percent. Click here for file

Additional file 8 **Genes in the *A. caulinodans *genome dedicated to transport**. From left to right: unique ID for each *A. caulinodans *gene; indication of the gene function based on homology; functional classification of genes according to Riley [26]; position of start codon or stop codon of a gene on the plus or minus strand, respectively; position of stop codon or start codon of a gene on the plus or minus strand, respectively; coding sequence on the plus or minus strand. Click here for file
