# Supplementary figures and images for: The genome of the versatile nitrogen fixer Azorhizobium caulinodans ORS571
Source: BMC Genomics. 2008 Jun 4;9:271. doi: 10.1186/1471-2164-9-271 (PMC2443382; doi:10.1186/1471-2164-9-271)

*Azorhizobium caulinodans* ORS571

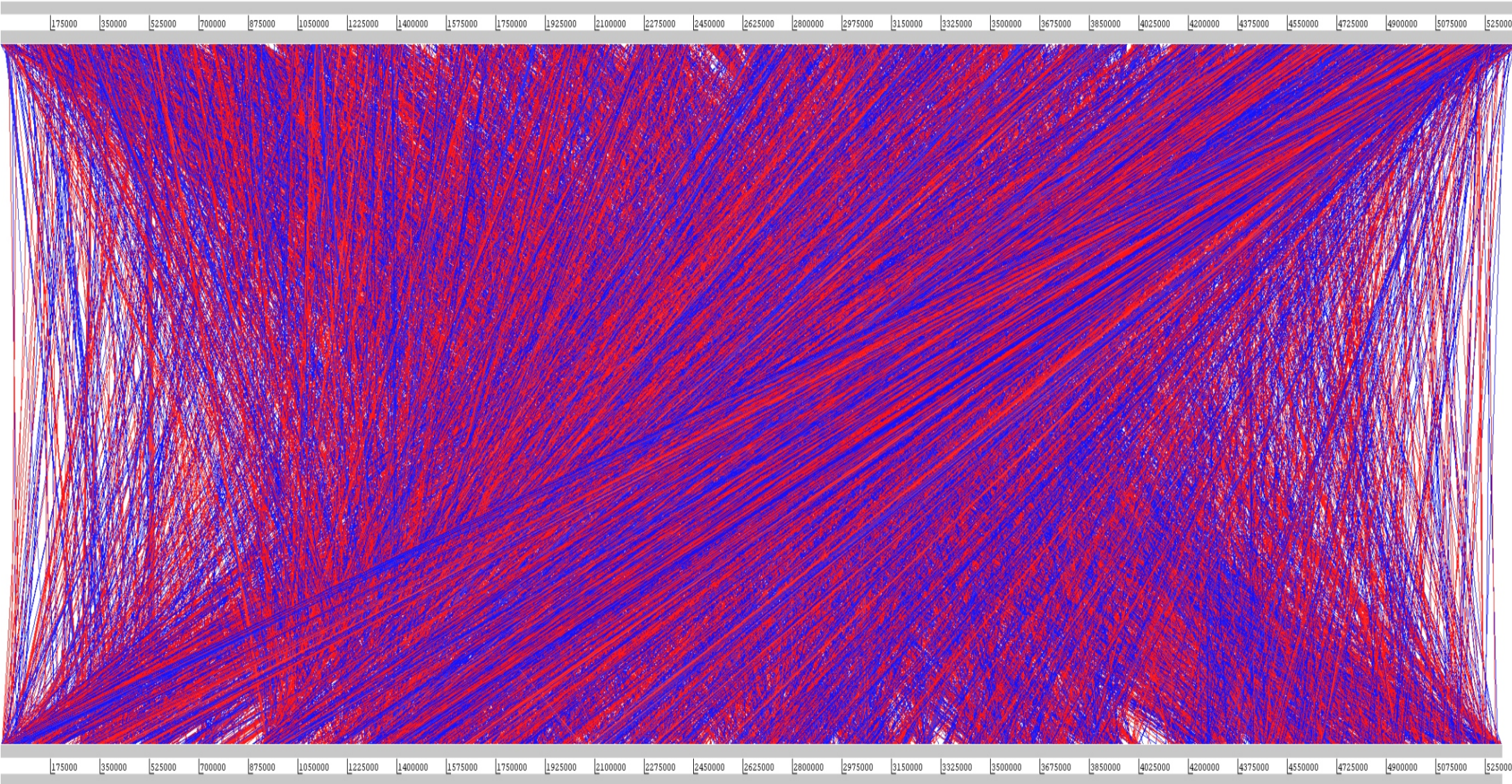

*Xanthobacter autotrophicus* Py2

Supplement: Additional file 3 — Whole genome comparison of A. caulinodans ORS571 and Xanthobacter autotrophicus Py2 using the ARTEMIS Comparison Tool [25]. Red and blue lines connect similar sequences and similar sequences that are inverted between strains, respectively. [file 1471-2164-9-271-S3.pdf]
